# Electric Field-Driven Modulation of Nanomechanical Interactions Between Tyrosine Kinase Inhibitors and Human Serum Albumin: Insights from AFM-Based Force Spectroscopy

**DOI:** 10.3390/molecules30173558

**Published:** 2025-08-30

**Authors:** Yuna Fu, Jianhua Wang, Di Gu, Letian Zhang

**Affiliations:** Key Laboratory of Biorheological Science and Technology, Ministry of Education, College of Bioengineering, Chongqing University, Chongqing 400044, China; fuyuna@cqu.edu.cn (Y.F.); gudi@stu.cqu.edu.cn (D.G.); 202419131092@stu.cqu.edu.cn (L.Z.)

**Keywords:** electric field, HSA, tyrosine kinase inhibitors, AFM, interaction

## Abstract

Electric fields are emerging as powerful tools to actively regulate biomolecular interactions at biointerfaces. In this study, we investigated how varying electric field strengths (0–100 mV/mm) influence the interfacial interaction between human serum albumin (HSA) and six tyrosine kinase inhibitors (TKIs): imatinib, bosutinib, dasatinib, nilotinib, ponatinib, and radotinib. Using atomic force microscopy (AFM), we quantified changes in adhesion force, specific (*F_i_*) and non-specific (*F*_0_) force, friction behavior, and protein morphology. Increasing field strength led to significant reductions in adhesion force (22–47%), *F_i_* (27–44%), *F*_0_ (38–53%), friction force (38–67%) and constant-load friction force (43–54%), along with decreased protein average surface height and roughness, indicating electric field-induced molecular compaction and interface smoothing. Notably, more hydrophobic TKIs showed greater responsiveness. These findings highlight the potential of electric fields to modulate protein–drug interactions in a controllable manner, offering a new strategy for the development of electrically tunable drug delivery systems and smart biomedical interfaces.

## 1. Introduction

Human serum albumin (HSA), the most abundant plasma protein in the human circulatory system, plays a pivotal role in the transport, distribution, and bioavailability of numerous endogenous and exogenous compounds, including pharmaceutical agents [1]. Among these, tyrosine kinase inhibitors (TKIs) represent a crucial class of targeted anticancer drugs, widely used in the treatment of chronic myeloid leukemia (CML) and other malignancies [2,3]. TKIs such as imatinib, bosutinib, dasatinib, nilotinib, ponatinib, and radotinib differ significantly in their chemical structure, hydrophobicity, and binding affinity, which in turn modulate their interactions with plasma proteins and affect their pharmacokinetics and therapeutic efficacy [4,5].

Understanding the interaction between TKIs and HSA at the molecular level is therefore essential for optimizing drug delivery, minimizing off-target effects, and designing next-generation TKIs with improved bioavailability. Previous studies have investigated the thermodynamic, spectroscopic, and docking characteristics of TKI–HSA complexes [6,7]. However, these approaches typically examine equilibrium properties and lack direct insight into the mechanical and morphological features of protein–drug interactions under physiologically relevant dynamic conditions.

Importantly, external physical stimuli such as electric fields are increasingly recognized as regulators of biomolecular interactions and structures [8]. Electric fields can alter protein conformation, affect hydration layers, disrupt electrostatic interactions, and modify surface adhesion and friction properties [9,10]. Despite growing interest, the influence of electric field strength on protein–drug interfacial interactions remain largely unexplored, particularly at the nanoscale.

To address this gap, the present study systematically investigates the effect of increasing electric field strength (0–100 mV/mm) on the interfacial interaction between HSA and six clinically relevant TKIs. Using atomic force microscopy (AFM)-based force spectroscopy and topographic imaging, we quantify changes in adhesion forces, specific and non-specific binding interactions (*F_i_*, *F*_0_), frictional behavior, and protein surface morphology (average surface height and roughness). This multifaceted approach provides a detailed mechanistic understanding of how electric fields modulate protein–drug interactions at the molecular interface. The findings offer new perspectives for developing electrically responsive drug delivery systems and enhancing the functional control of biointerfaces in nanomedicine.

## 2. Results and Discussion

### 2.1. Morphological Changes of HSA Induced by Electric Fields

**Figure 1:**
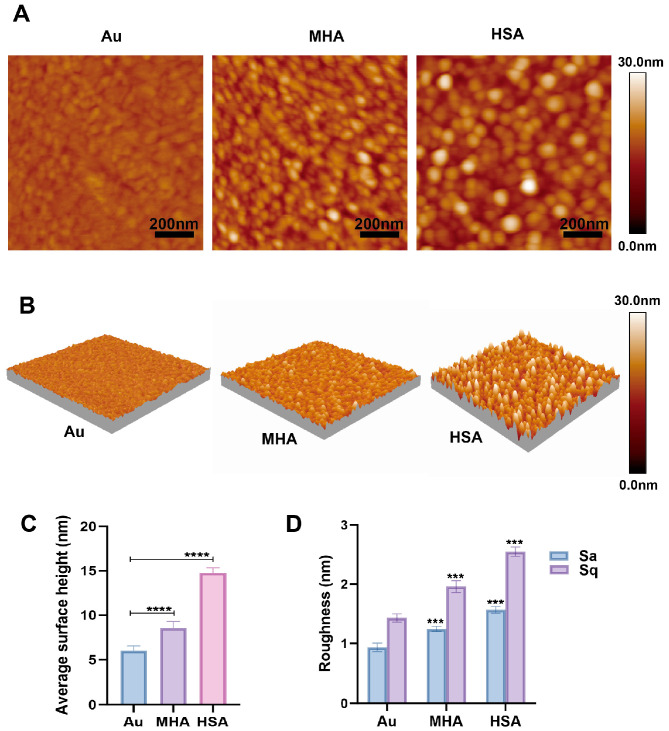
AFM characterization of bare gold substrate (Au), MHA self-assembled monolayer, and human serum albumin (HSA)-immobilized surfaces. (**A**) 2D AFM topographic images (scan size: 1 µm × 1 µm) of Au, MHA layer, and HSA-functionalized surfaces. (**B**) Corresponding 3D AFM topographic renderings highlighting surface morphology variations among Au, MHA, and HSA layers. (**C**) Average surface height and (**D**) surface roughness parameters (Sa: average roughness; Sq: root-mean-square roughness) derived from AFM images. Data are presented as mean ± standard deviation. (ns: not statistically significant, *** indicates *p* < 0.001, **** indicates *p* < 0.0001).

Among the various testing modes of AFM, the tapping mode is more suitable for determining the morphology of soft samples and does not require staining or labeling. Therefore, we used the tapping mode of AFM for protein imaging to assess the morphology of proteins under electric field stimulation. In the AFM testing, we first examined the morphological characteristics of the protein monolayer to determine whether the protein had successfully coupled to the gold substrate. As shown in Figure 1, the average surface height determined from AFM topographic profiles increased from 6.9 ± 0.1 nm for the bare gold substrate to 15.4 ± 0.1 nm after HSA immobilization. Here, average surface height denotes the mean height over the scanned area relative to the reference plane and does not represent the size of a single HSA molecule. Apparent height values can be larger than single-molecule dimensions due to orientation, aggregation, conformational variability, and hydration layers [11,12]. Meanwhile, the root mean square (RMS) value of roughness increased from 1.4 ± 0.1 nm (gold substrate) to 2.6 ± 0.1 nm (HSA layer), indicating that the protein layer exhibited slightly higher surface unevenness. Additionally, from the 2D (Figure 1A) and 3D topography images (Figure 1B), it is clearly observed that HSA particles are uniformly distributed on the gold substrate. These results indicate that the HSA molecular layer was successfully prepared.

**Figure 2:**
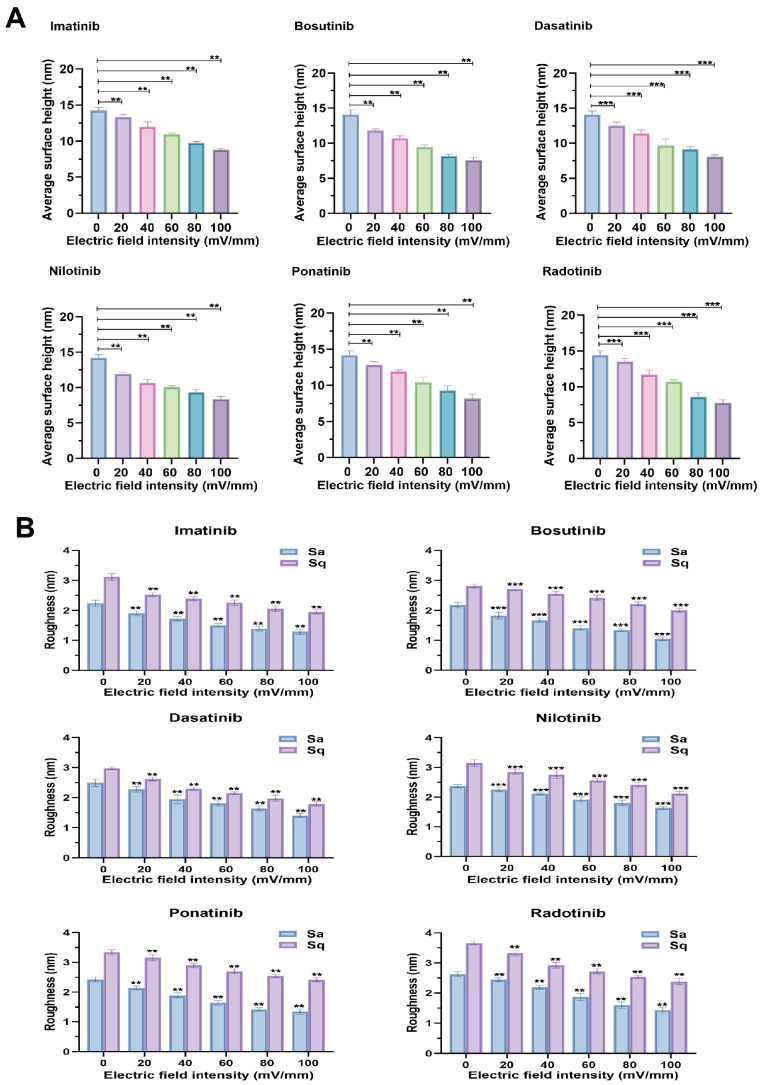
Quantitative analysis of the surface morphology of HSA-HSA in the presence of TKI drugs under different electric field strengths (0–100 mV/mm) using atomic force microscopy (AFM). (**A**) Average surface height variations for HSA in the presence of six TKIs (imatinib, bosutinib, dasatinib, nilotinib, ponatinib, radotinib) at increasing electric field intensities. (**B**) Surface roughness parameters Sa (average roughness) and Sq (root mean square roughness) for the same systems. Statistical significance compared with 0 mV/mm: ns: not statistically significant, ** indicates *p* < 0.005, *** indicates *p* < 0.001, Each bar chart represents data measured based on 50 AFM topography scans under each condition, with data presented as mean ± standard deviation.

Under the influence of an electric field, the interactions between proteins and drugs undergo various changes. Among these, alterations in the average surface height and roughness of proteins serve as a direct reflection of the electric field’s impact, thereby indicating changes in protein properties. Figure 2 illustrate the evolution of protein conformation (average surface height) and surface morphology (roughness) of HSA–drug complexes under different electric field intensities in six TKI systems. The results consistently show that increasing electric field strength induces compaction and surface smoothing of HSA films, modulating the nanoscale structure of the protein–drug interface.

As shown in Figure 2A, under an electric field strength of 0 mV/mm, the average surface height of HSA molecules measured by atomic force microscopy (AFM) ranged from 14.2 ± 0.1 nm (dasatinib–HSA) to 15.3 ± 0.1 nm (bosutinib–HSA). When the electric field strength was increased to 100 mV/mm, the average surface height of HSA in all systems decreased significantly. Imatinib decreased from 14.6 ± 0.1 nm to 8.6 ± 0.2 nm, while the average surface height reduction rates for nilotinib and ponatinib were approximately 42% and 44%, respectively. This progressive decrease indicates that the electric field promotes structural compression of the HSA–drug complex layer on the substrate surface. Consistent with this trend, roughness decreased uniformly with increasing electric field strength (Figure 2B), indicating that the external electric field induced conformational smoothing of the HSA–drug complex and more uniform arrangement on the substrate. At 0 mV/mm, the initial average roughness values (sa) ranged from 2.1 ± 0.1 nm (bosutinib–HSA) to 2.7 ± 0.1 nm (radotinib–HSA). At 100 mV/mm, the roughness significantly decreased, with nilotinib decreasing from 2.4 ± 0.1 nm to 1.6 ± 0.1 nm, (a reduction rate of approximately 31%), while imatinib and ponatinib had reduction rates of approximately 43% and 44%, respectively. These trends indicate that external electric fields drive nanoscale rearrangement of HSA–drug complexes, resulting in vertical compression (average surface height reduction) and lateral smoothing (roughness reduction). Mechanistically, this effect may arise from electric field-induced reorientation of protein domain dipoles, promoting molecular flattening and tighter packing on the substrate [13,14]. Additionally, the suppression of hydrophobic domain aggregation under the electric field, particularly for highly hydrophobic tyrosine kinase inhibitors (TKIs) such as radotinib and nilotinib, results in stronger compression and surface smoothing.

### 2.2. Adhesion Force Characterization Under Electric Fields

**Figure 3:**
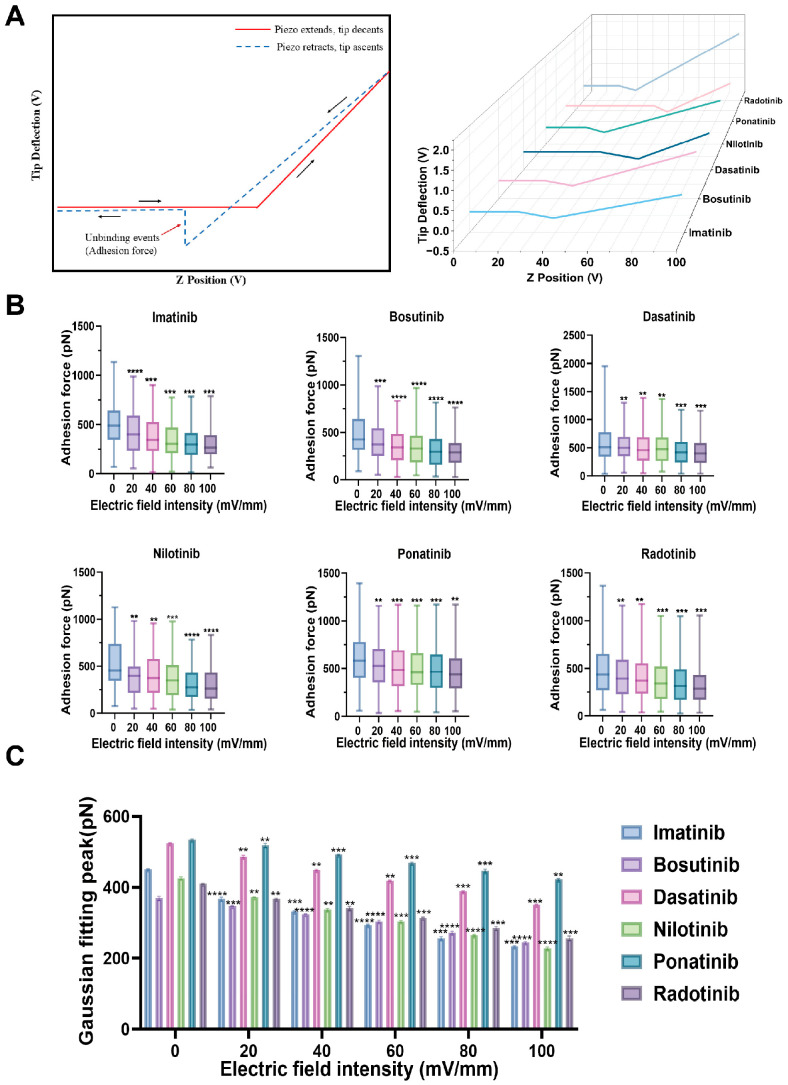
Measurement of adhesive forces between HSA molecules in different TKI solutions at different electric field strengths. (**A**) Left panel: Schematic representation of an AFM force–distance curve illustrating the measurement of adhesion forces. The approach segment (red) corresponds to the cantilever’s movement toward the sample surface, while the retraction segment (blue) records the separation process. Adhesion is the maximum attractive force observed during the retraction process, obtained from the unbinding event. Right panel: Representative retraction force–distance curves obtained for HSA–HSA interactions in the presence of six tyrosine kinase inhibitors (TKIs) under 20 mV/mm electric field conditions. Adhesion forces were extracted from the unbinding events. (**B**) Box plot of adhesion forces of HSA and six TKIs under electric field intensity (0–100 mV/mm). Each box plot represents the statistical distribution of 300 force–distance curves. (**C**) Perform Gaussian fitting on the adhesion histogram and extract the peak force values under the same conditions. The peak value represents the most likely adhesion force in each case. The error bar represents the standard deviation. (ns: not statistically significant, ** indicates *p* < 0.005, *** indicates *p* < 0.001, **** indicates *p* < 0.0001).

Proteins undergo a series of conformational changes under the influence of an external electric field, thereby altering their function and properties [9]. Consequently, the interactions between proteins and drugs also change accordingly. To evaluate how electric fields influence the interfacial binding behavior of serum proteins in drug environments, we first measured the adhesion forces between HSA molecules in the presence of six different tyrosine kinase inhibitors (TKIs, imatinib, bosutinib, dasatinib, nilotinib, ponatinib, radotinib) under varying electric field strengths (0–100 mV/mm) using atomic force microscopy (AFM). Figure 3A (take the example of an electric field of 20 mV/mm.) shows the F–D curve obtained during the measurement of adhesion force. Based on the F–D curve, sufficient adhesion force data values were obtained, which were used for two complementary analyses: displaying the statistical distribution of adhesion force values via a box plot (Figure 3B), and performing a Gaussian fitting on the force histogram to extract the most likely adhesion force values (Figure 3C).

As shown in Figure 3B, after applying an electric field, the adhesive force between HSA molecules decreased in the presence of tyrosine kinase inhibitors (TKIs). At the same time, we found that the adhesive force decreased with increasing electric field strength (Figure 3C). For example, in the bosutinib group, adhesion force decreased from 347 ± 1 pN at 20 mV/mm to 244 ± 3 pN at 100 mV/mm. Nilotinib (372 ± 2 pN to 228 ± 4 pN) and radotinib (367 ± 2 pN to 256 ± 6 pN) also showed a similar trend. As the electric field strength increased, the decrease in interquartile ranges (IQRs) indicated reduced force variability, which may be due to electric field-induced HSA denaturation or conformational reorientation, resulting in a reduction in accessible binding epitopes [15,16]. The extent of adhesion force reduction varies significantly among different TKIs. Notably, imatinib (~36% reduction) and nilotinib (~39% reduction) exhibit a marked decrease, indicating that their binding relies on electrostatic and polar interactions, which become more unstable under the influence of an electric field [17,18]. In contrast, ponatinib exhibited the smallest change (peak force decrease of approximately 19%), suggesting that hydrophobic interactions or deep binding conformations are less affected by external fields and are the primary contributing factors. This finding is supported by previous docking and binding studies, which indicate that ponatinib tends to occupy deeper pockets in serum albumin with limited surface exposure [19].

In summary, these results collectively indicate that increased electric field strength significantly reduces the adhesion force between HSA molecules in all tyrosine kinase inhibitor (TKI) systems, suggesting that electric field-induced weakening of intermolecular binding forces may be achieved by altering protein conformation or binding orientation [20]. These findings suggest a potential strategy for selectively modulating protein–drug interactions using electric fields.

### 2.3. Specific and Non-Specific Force Analysis via Poisson Modeling

To further dissect the molecular interactions underlying the observed adhesion force changes, we applied Poisson analysis to distinguish specific (*F_i_*) and non-specific (*F*_0_) force, enabling a more mechanistic understanding of how electric fields modulate HSA–TKI binding events at the single-molecule level. According to this model (Formula (1)), the variance (*σ*^2^*_m_*) of the force distribution should exhibit a linear relationship with the average adhesion force (*μ_m_*), where the slope represents the specific force *F_i_*, and the intercept corresponds to −*F_i_·F*_0_, with *F*_0_ denoting the non-specific force component [21,22].

*σ*^2^_*m*_ = *μ*_*m*_*F*_*i*_ − *F*_*i*_*F*_0_ (1)

**Figure 4:**
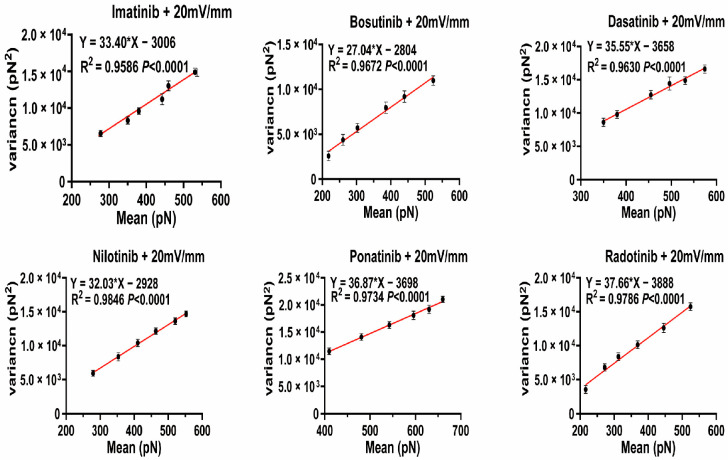
Linear fitting analysis of the relationship between the mean adhesion force (*μ_m_*) and the force variance (*σ*^2^*_m_*) for HSA molecules in the presence of six different TKIs (20 mV/mm), measured by AFM-based force spectroscopy. Each data point represents the average value obtained from multiple force–distance curves, and error bars indicate the standard deviation. The fitted lines were analyzed based on the Poisson model to separate the specific force (*F_i_*) and non-specific force (*F*_0_).The corresponding R^2^ values and fitted equations are shown in the figure.

As shown in Figure 4 (using 20 mV/mm electric field as an example), there is a strong linear correlation between the variance and average adhesion force of all tested TKI–HSA systems (R^2^ > 0.95), confirming the applicability of the Poisson model in describing dissociation behavior. For example, in the case of the imatinib–HSA interactions, linear regression yielded a slope *F_i_* = 33.40 ± 0.38 pN, representing the most likely specific force per molecular binding event. The non-specific force *F*_0_ was calculated from the intercept to be approximately 90 ± 2 pN. Similar analyses for other TKIs show that the *F_i_* values range from 27.0 ± 0.4 pN to 37.7 ± 0.5 pN, and the *F*_0_ values range from 91.4 ± 0.9 pN to 103.7 ± 0.9 pN, depending on the drug molecule (Table 1). These values fall within the expected range of single-molecule protein ligand breakage forces reported in the literature [23,24], thereby validating our AFM-based force spectroscopy method. Interestingly, in earlier analyses, drugs with higher *F_i_* values (e.g., dasatinib, *F_i_* = 35.6 ± 0.7 pN) also exhibited stronger elasticity in electric field-induced dissociation (Figure 3), suggesting a correlation between binding strength and electric field stability. Meanwhile, higher *F*_0_ values may reflect a broader molecular contact area or increased non-specific adhesion due to surface conformational rearrangement [25].

**Figure 5:**
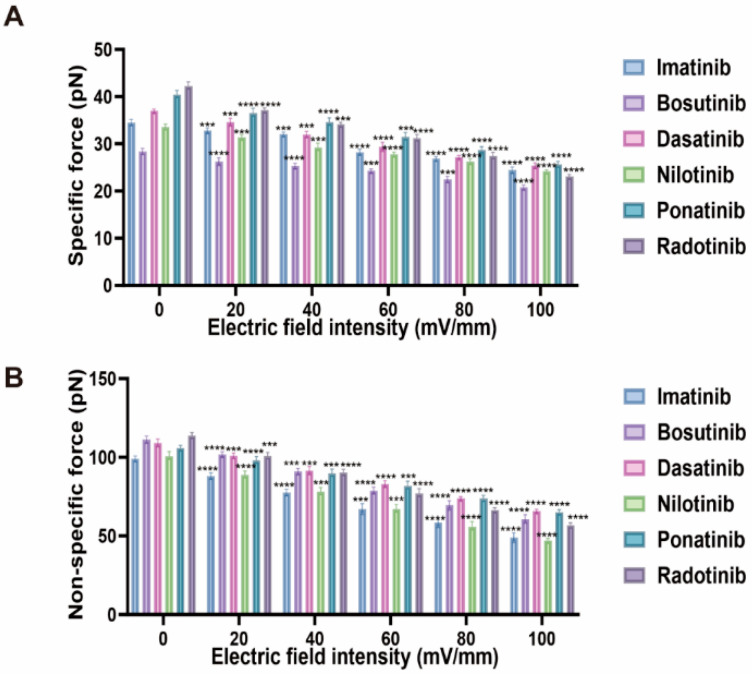
Electric field dependence of specific (*F_i_*) and non-specific (*F*_0_) force between human serum albumin (HSA) molecules in tyrosine kinase inhibitor (TKIs) solution. The data were collected under electric fields of 0, 20, 40, 60 80 and 100 mV/mm. The force value was obtained by fitting the force-distance curve using Gaussian fitting. The data represents the average ± standard deviation of the Gaussian peak positions obtained from more than 300 independent measurements under each condition. (ns: not statistically significant, *** indicates *p* < 0.001, **** indicates *p* < 0.0001).

Figure 5 comprehensively illustrates the modulation of specific forces (*F_i_*) and non-specific force (*F*_0_) between human serum albumin (HSA) molecules under different electric field strengths (0–100 mV/mm) in the presence of various tyrosine kinase inhibitors (TKIs). Upon application of an electric field stimulus, both *F_i_* and *F*_0_ exhibited a decreasing trend in all tested drugs, and this decrease showed a systematic weakening trend as the electric field strength increased; however, the magnitude and pattern of this weakening varied between specific and non-specific interaction forces and among different TKI molecules.

The specific force (*F_i_*) reflects the direct binding strength between the TKI molecule and the drug-binding sites of HSA, exhibiting the highly significant sensitivity to electric field strength. At 20 mV/mm, radotinib exhibited the highest *F_i_* value (37.7 ± 0.5 pN), followed by ponatinib (36.9 ± 0.5 pN), dasatinib (35.6 ± 0.7 pN), imatinib (33.4 ± 0.4 pN), nilotinib (32.0 ± 0.7 pN), and bosutinib (27.0 ± 0.4 pN). When the electric field strength was increased to 100 mV/mm, the *F_i_* values decreased by 21–37%, with radotinib showing the largest decrease (to 23.6 ± 0.4 pN, ~37% decrease), while ponatinib maintained a relatively high *F_i_* value (25.4 ± 0.6 pN, ~31% decrease). This indicates that specific binding interactions are influenced by the electric field, a phenomenon similar to previous studies by Ma et al., that is, the external electric fields can disrupt ligand-protein complexes by disturbing dipole–dipole interactions and altering the energy landscape of the binding pocket [26,27]. Mechanistically, electric fields may induce conformational rearrangements within HSA’s ligand-binding domain IIA [28], thereby altering hydrogen bond networks and electrostatic interactions critical for high-affinity drug binding. In addition, the heterogeneous electrostatic potential on the HSA surface—originating from positively charged lysine/arginine and negatively charged aspartate/glutamate residues—plays a key role in ligand recognition. External electric fields can redistribute these surface charges, modulating the local potential landscape of the binding pocket. Likewise, the TKIs used in this study possess distinct charge distributions and dipole moments, which determine their electrostatic complementarity with HSA; under field perturbation, this complementarity can be reduced, weakening *F_i_* [1,29]. Additionally, field-induced polarization of the TKI molecules themselves may reduce complementarity with the binding pocket, as has been reported in molecular dynamics simulations of ligand–protein complexes under field perturbation [30].

Non-specific forces (*F*_0_), which primarily arise from van der Waals forces, hydrophobic interactions, and transient contacts during unbinding [31,32]. When an electric field is applied, *F*_0_ decreases overall and shows a decreasing trend as the electric field strength increases. At an electric field strength of 20 mV/mm, the *F*_0_ values ranged from 90 ± 2 pN (imatinib) to 103.7 ± 0.9 pN (bosutinib). When the electric field strength was increased to 100 mV/mm, these values decreased to 52 ± 1 pN and 64 ± 1 pN, respectively. The percentage decrease in *F*_0_ (ranging from 33% to 45%) indicates that the electric field significantly weakens the non-specific force between HSA and TKIs. The higher *F*_0_ values in radotinib and nilotinib systems likely result from stronger hydrophobic and van der Waals interactions between drug-bound HSA surfaces, which contribute significantly to non-specific force [33,34]. From an electrostatic perspective, the long-range interaction component of F0 can also be influenced by the net surface charge of HSA and the electrostatic potential of the drug molecule. Redistribution of surface charges under an electric field can alter hydration layer structuring and screening effects, indirectly reducing the magnitude of hydrophobic-driven non-specific adhesion [29]. Notably, drugs with initially stronger hydrophobic interactions (radotinib, nilotinib) exhibited larger absolute reductions in *F*_0_ under electric fields, confirming that hydrophobic-driven non-specific forces are particularly sensitive to electric field modulation. In contrast, the interaction of dasatinib relies more on hydrogen bonds and electrostatic forces, showing smaller relative changes, which is consistent with the more stable interaction pattern under the external electric field [35,36].

In conclusion, an increase in electric field strength systematically weakens both the specific and non-specific interaction forces between HSAs, with the most significant effect observed on non-specific binding force (*F*_0_). The extent of this modulation varies among different TKIs, reflecting differences in their physicochemical binding mechanisms. These findings suggest that electric fields can serve as a powerful tool for detecting and potentially modulating drug-protein interactions, with significant implications for regulating pharmacokinetics and drug release characteristics in biomedical applications [37,38].

### 2.4. Nanotribological Properties Under Electric Field Exposure

**Figure 6:**
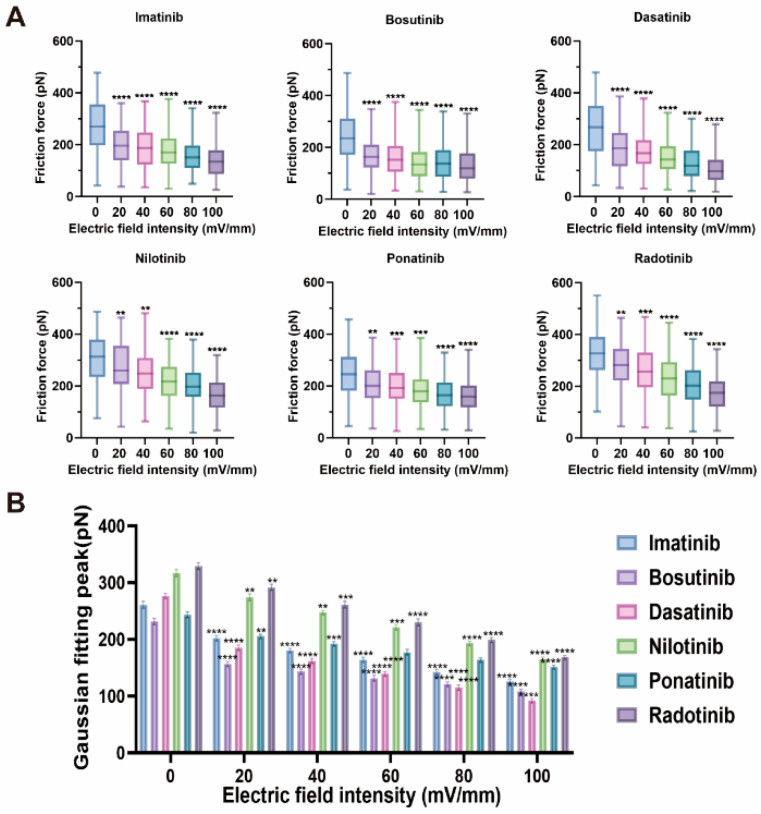
Trends of intermolecular friction forces of HSA in the presence of six TKIs (Imatinib, Bosutinib, Dasatinib, Nilotinib, Ponatinib, and Radotinib) under different electric field strengths (0, 20, 40, 60, 80, and 100 mV/mm), measured using atomic force microscopy (AFM). (**A**) Box plots of friction forces (n = 100 force curves per condition). (**B**) Trends of Gaussian peak values fitted from the distributions in (**A**), presented as mean ± standard deviation. Statistical significance: ns indicates no significant difference, ** indicates *p* < 0.005, *** indicates *p* < 0.001, **** indicates *p* < 0.0001.

Given the influence of electric fields on binding forces, we next examined how these fields affect frictional interactions at the HSA–TKI interface using loop friction mode in AFM. This nanotribological analysis reflects dynamic contact responses relevant to protein–drug interface stability. Figure 6 summarizes the influence of electric field strength on the intermolecular frictional forces of HSA in the presence of different TKI drugs, providing indirect insights into the regulation of drug–protein interaction dynamics. Friction forces were measured using atomic force microscopy (AFM), with their distribution analyzed via box plots (Figure 6A) and Gaussian-fitted peaks (Figure 6B). Among the six TKI drugs studied, it was observed that the overall friction force decreased upon the application of an electric field, and there was a trend of friction force decreasing with increasing electric field strength; however, the extent of decrease varied among different drugs.

Under a 20 mV/mm electric field, radotinib (292 ± 5 pN) exhibited the highest friction force peak, followed by nilotinib (274 ± 6 pN), ponatinib (206 ± 4 pN), imatinib (202 ± 5 pN), dasatinib (185 ± 5 pN), and bosutinib (157 ± 4 pN).These differences reflect variations in the strength of drug–protein interactions, influenced by drug hydrophobicity, molecular size, accessibility of binding sites, and interaction types (hydrophobic, electrostatic, π-π stacking) [39,40]. As the electric field increases, friction force exhibits a gradient decrease, with a significant reduction when the electric field strength reaches 100 mV/mm. For example, in the imatinib–HSA system, the friction force decreased from 202 ± 5 pN to 126 ± 4 pN, which amounts to a 38% reduction. It is speculated that this may be due to imatinib being hydrophobic and embedded in the HSA IIA site, while being stabilized by hydrogen bonds [41]. The electric field-induced protein conformation softening reduces the contact area and number of hydrogen bonds, thereby lowering the friction resistance [42]. In the dasatinib system, the friction force decreased from 185 ± 5 pN to 92 ± 3 pN, with the largest decrease of 50%. It is speculated that this may be because dasatinib primarily binds to HSA through electrostatic interactions [6], and the electric field effect reduces Coulombic attraction between proteins by shielding the electrostatic potential field, thereby promoting a decrease in friction force [43]. Additionally, radotinib and nilotinib, which initially exhibited the highest friction coefficients, also showed significant reductions, potentially due to their stronger hydrophobicity and π-π stacking interactions, which are particularly sensitive to electric field-induced conformational changes [44]. In contrast, ponatinib, which relies more on electrostatic and hydrogen bonding interactions (a ~26% reduction rate), showed a relatively smaller decrease, which may be related to the partial retention of electrostatic contacts under the influence of an electric field [45]. The decreasing loop friction forces with stronger electric fields indicate a reduction in interfacial energy dissipation, which likely arises from smoother topography, diminished molecular entanglement, and reduced adhesive hysteresis under field-modulated conditions [46,47].

### 2.5. Constant-Load Friction Force and Its Dependence on Electric Field Strength

**Figure 7:**
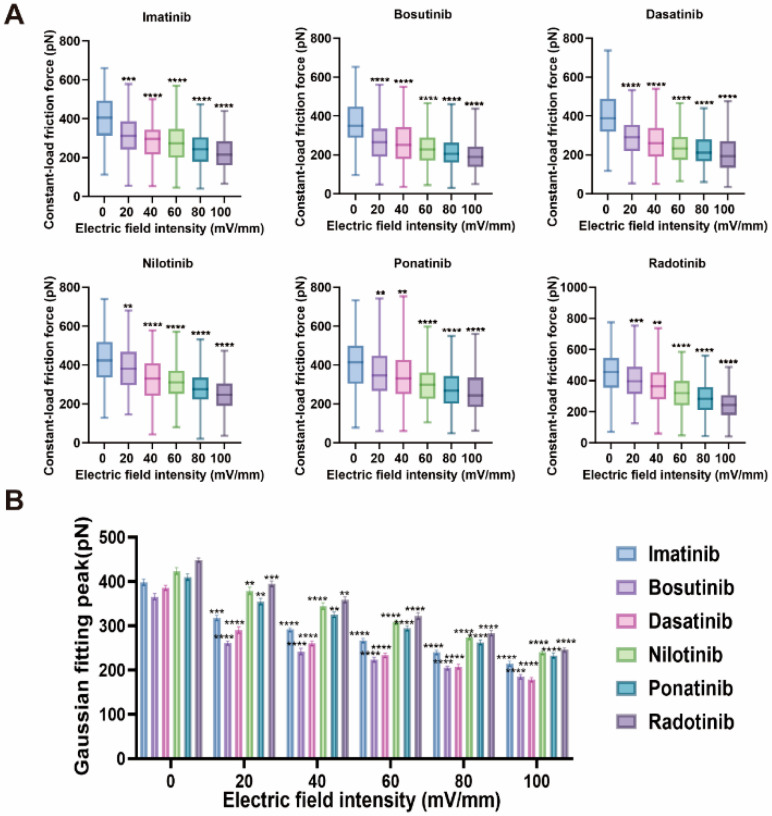
Comparison of constant-load friction forces between HSA molecules in the presence of six TKIs (Imatinib, Bosutinib, Dasatinib, Nilotinib, Ponatinib, and Radotinib) under different electric field strengths (0–100 mV/mm), measured using atomic force microscopy (AFM) at the constant load of 1 nN (n = 100 measurements per condition). (**A**) Box plots of constant-load friction forces. (**B**) Gaussian peak distributions fitted from the friction force data in (**A**), presented as mean ± standard deviation. Statistical significance: ns indicates no significant difference, ** denotes *p* < 0.005, *** denotes *p* < 0.001, **** denotes *p* < 0.0001.

In addition to loop friction, we investigated the impact of electric fields on steady-state frictional forces under constant normal load conditions, which more closely simulate sustained interactions in physiological or biomedical interface environments. Figure 7 shows the variation in constant load friction force between HSA molecules under different electric field strengths (0–100 mV/mm), covering six TKI drug systems. These measurement results reflect the lateral resistance encountered by the HSA layer when a constant load of 1 nN is applied, further revealing how the electric field modulates the mechanical properties at the protein–drug interface.

We found that under zero electric field conditions, radotinib exhibited the highest friction force of 448.0 ± 4 pN, followed by nilotinib (423 ± 8 pN), ponatinib (410 ± 7 pN), imatinib (398 ± 7 pN), dasatinib (386 ± 5 pN), and bosutinib (365 ± 7 pN).These trends align with the initial hydrophobic interactions and π-π interactions between the drug molecules and the HSA surface [34], which dominate energy dissipation during the friction process. When an electric field is applied, the constant load friction force between HSA molecules in the TKI solution exhibits a decreasing trend. As the electric field strength increases from 20 mV/mm to 100 mV/mm, all systems show a significant and consistent decrease in friction force. Among them, imatinib has moderate hydrophilicity (logP = 3.1), and its friction behavior is primarily determined by electrostatic interactions and hydrogen bonding interactions [48]. Under the influence of the electric field, these interactions are partially disrupted, leading to dipole reorientation and hydration layer expansion [49], resulting in a reduction of approximately 33% in constant load friction force. Ponatinib (logP = 4.5) exhibits stronger hydrophobicity and π-π stacking interactions, resulting in higher initial friction force. These hydrophobic interactions are particularly sensitive to electric field-induced hydration and conformational rearrangement, explaining 34% of the friction force decrease [19]. Nilotinib decreased from 379 ± 8 pN to 240 ± 4 pN (a ~37% reduction rate), while radotinib decreased from 395 ± 6 pN to 246 ± 4 pN (a ~38% reduction rate). This can be attributed to the effective suppression of extensive van der Waals and hydrophobic interactions by electric field-induced protein unfolding and hydration lubrication [50]. Additionally, the observed reduction in friction force of 29–39% under constant load indicates that most of the original interface load-bearing capacity is lost due to electric field-induced molecular rearrangement [51], a phenomenon that does not occur under no load or pure adhesion measurements (only *F_i_* or *F*_0_). This supports the hypothesis that external electric fields regulate the mechanical and interface friction properties of protein–drug complexes in a load-dependent manner.

## 3. Materials and Methods

### 3.1. Materials

Tyrosine kinase inhibitors (imatinib, bosutinib, dasatinib, nilotinib, ponatinib, radotinib) were all sourced from MedChemExpress (Shanghai, China). Human serum albumin (HSA),16-mercaptohexadecanoic acid (MHA), 1-ethyl-(3-dimethylaminopropyl)carbodiimide (EDC), and *n*-hydroxy succinimide (NHS) were purchased from Sigma-Aldrich (Saint Louis, MO, USA). Gold-coated silicon wafers were purchased from Ted Pella Inc. (CA, USA), and AFM probes of different models were purchased from BudgetSensors (Sofia, Bulgaria). Phosphate-buffered saline (10 mM PBS, 140 mM NaCl, 3 mM KCI, pH 7.4), ethanol (HPLC grade ≥ 99.8%) and Dimethyl sulfoxide were supplied by MACKLIN (Shanghai, China) All reagents are analytical grade and can be used without further purification.

### 3.2. Pretreatment of the Substrates

We chose to modify the gold substrate surface using sulfur-containing molecules with strong affinity for gold, forming a layer of MHA molecules containing thiols to facilitate the subsequent fixation of proteins on the substrate. First, the gold-plated silicon substrate (1 cm × 1 cm) was rinsed three times with ethanol and ultra-pure water. Then, H_2_O_2_ was slowly added to H_2_SO_4_ solution and mixed to prepare a highly corrosive piranha solution (H_2_SO_4_:H_2_O_2_ = 3:1, *v*/*v*). After cooling, the substrate from the previous step was immersed in this solution for 30 min to further remove organic contaminants from the substrate surface. The washed substrate is then rinsed three times with ultra-pure water and anhydrous ethanol alternately, and dried with nitrogen gas. Finally, the cleaned gold surface was placed in a 1 mM MHA ethanol solution, incubated at room temperature for 24 h, then sonicated in ethanol for 3 min to remove unreacted MHA molecules. The substrate was then rinsed three times with anhydrous ethanol and ultrapure water alternately and immediately dried with nitrogen to form a stable MHA molecular layer.

### 3.3. Preparation of the Protein Molecular Layer

To conjugate proteins to the gold substrate, the terminal carboxyl groups of the prepared MHA molecular layer must be activated. Specifically, at room temperature, the MHA molecular layer was immersed in a PBS buffer solution containing NHS (2 mg/mL) and EDC (2 mg/mL) mixed in a 1:1 ratio and incubated for 1 h. The activated layer was then rinsed with a large amount of ultrapure water to remove free EDC and NHS molecules and then dried under a nitrogen stream. It was then immediately placed in a 40 mg/mL HSA solution and incubated at 4 °C for 12 h. The incubated protein layer was gently rinsed with ultrapure water and dried with nitrogen, and the cured protein layer was finally reacted in a TKIs solution for 12 h. All TKIs were dissolved in DMSO/PBS mixture to prepare a 1 mM stock solution, which was then diluted with PBS solution to a working solution concentration of 3 μM. The prepared protein layer and diluted working solution were used immediately to avoid contamination of the protein layer and drug solution. The AFM gold-coated probes were modified using the same method described above.

### 3.4. Electric Field Stimulation of Varying Intensities

In this study, a homemade in vitro electric stimulation device (Figure S1) was used to explore the effect of an applied electric field on the interaction between HSA and TKI drugs. A pair of stimulation electrodes were inserted through the lid of a cell culture dish with a diameter of 15 cm and positioned vertically relative to the bottom of the dish, which had a height of 25 mm. The sample was placed at the center of the stimulation electrodes, with a distance of 6.5 cm between the positive and negative electrodes, which were kept parallel. The electrodes were made of platinum metal, with dimensions of 10 × 20 × 0.2 mm. The electrodes were immersed in a drug solution at room temperature, and a constant electric field with different voltages (0, 20, 40, 60, 80, 100 mV/mm) was applied to the experimental samples via an external power source. After 1 h of stimulation, the samples were immediately placed in a liquid pool and analyzed using AFM for morphological and mechanical characterization.

### 3.5. AFM Measurement of Protein Layer Morphology

All protein imaging and mechanical measurements were performed using a JPK Nanowizard II atomic force microscope (Berlin, Germany). In topography measurements, the experiment used a probe (Tap150AI-G) with a force constant of 5 N/m and a resonance frequency of 150 kHz, featuring a tip height of 17 μm, a radius of 10 nm, and a tip half-cone angle of 10°. The experiment employed AFM tapping mode with a scanning range of 1 μm × 1 μm and a resolution of 512 × 512 pixels. Additionally, feedback parameters (gain, reference point setting, and drive frequency) were optimized to minimize direct damage to the proteins caused by the applied force. We analyzed the AFM topography images using Image 4.7 software to obtain two quantitative parameters: (1) average surface height, which refers to the average height of the scanned surface area calculated from the height contour of the AFM image, and which was obtained from AFM topography images via cross-sectional analysis, and (2) the RMS roughness (root mean square), which characterizes the surface evenness of both the bare substrate and the protein layer.

### 3.6. AFM Measurement of Protein Mechanical Properties

AFM contact mode was used to investigate changes in protein adhesion force following electrical stimulation. The experiments were conducted at room temperature using a gold-coated probe of model number ContGB-G (force constant 0.2 N/m, resonant frequency 13 kHz). The sample was scanned at a frequency of 1 Hz, with a scanning range of 1 × 1 cm^2^ and a resolution of 512 × 512 pixels. During the measurement process, at least 300 force curves were measured for each sample. All force curves were analyzed using JPKSPM data processing software (JPK, version 7.0.97). According to Hooke’s law F = kd, the voltage-distance curves recorded by AFM can be converted into force-distance curves, thereby obtaining the adhesion force values. In this study, adhesion force refers to the unbinding events observed during the retraction segment of the F–D curve, corresponding to the maximum attractive force required to separate the AFM tip and the sample surface after contact. The specific interaction forces (*F_i_*) and non-specific interaction forces (*F*_0_) were determined by fitting the force–distance curve distributions with the Poisson step analysis method. In this context, “specific” refers to reproducible adhesion events attributable to interactions between HSA molecules immobilized on the AFM tip and substrate under the given AFM configuration, rather than selective molecular recognition in vivo. This definition is consistent with established AFM-based single-molecule force spectroscopy terminology [52,53].

Friction force measurements were performed using gold-plated probes of the same specifications and under the same experimental conditions in the friction force mode (FFM) of the AFM. At least 100 measurement curves were recorded for each sample. The friction force is calculated from the friction loop by recording the lateral voltage signal of the cantilever as it slides across the substrate, where the signal equals half the difference between the lateral voltage values obtained in the forward and reverse scanning directions (*V_f_*).

(2) $$V_{f}\;=\frac{V_{\mathrm{trace}}-V_{\mathrm{retrace}}}{2}$$

Further conversion of voltage values into friction force [54]

(3) $$F_{f\;}=\frac{3}{2}K_{f}\frac{h}{l}SV_{f}$$

In the above equation, *K_f_* is the lateral spring coefficient of the cantilever, *h* is the tip height, *l* is the cantilever length, and *S* is the deflection sensitivity of the detector.

### 3.7. Data Statistics

Force curves data were statistically analyzed and summarized using Microsoft Office Home and Student Excel 2019 (Microsoft, Redmond, WA, USA). For each experimental condition, more than 300 independent force–distance curves were collected from spatially distinct locations on the HSA-functionalized gold substrate. Gaussian fitting of force data was performed using the nonlinear curve fitting module of OriginPro 2019 (OriginLab, Northampton, MA, USA), and the values from the Gaussian peak positions were reported as mean ± standard deviation (SD). This fitting procedure reduces the influence of outlier events and reflects the central tendency of the interaction force distribution. Statistical significance was calculated using an unpaired t-test performed with GraphPad Prism version 10.1.2 (GraphPad Software, La Jolla, CA, USA) to calculate statistical significance. In this paper, “ns” denotes “not statistically significant,” while * *p* < 0.05, ** *p* < 0.01, *** *p* < 0.001, and **** *p* < 0.0001 indicate statistically significant differences.

## 4. Conclusions

In this study, we systematically investigated the effect of external electric fields on the interactions between human serum albumin (HSA) and six representative tyrosine kinase inhibitors (TKIs, imatinib, bosutinib, dasatinib, nilotinib, ponatinib, and radotinib) using atomic force microscopy (AFM)-based force spectroscopy and topographical imaging. By combining adhesion force analysis, specific (*F_i_*) and non-specific (*F*_0_) forces derived from the Poisson method, friction force measurements, and morphological characterization (average surface height and surface roughness), we provide a comprehensive picture of how electric fields modulate the mechanical and structural properties of the HSA–drug interface.

A consistent observation across all measurements was that protein–protein interactions gradually weakened as electric field strength increased (0–100 mV/mm), indirectly indicating enhanced interactions between proteins and drugs. Specifically, under electric field stimulation, both adhesion force and the *F_i_*/*F*_0_ component decreased significantly, with *F_i_* decreasing by 27–39% and *F*_0_ decreasing by 38–53%. These results indicate that electric fields can disrupt specific hydrogen bonds and hydrophobic interactions, as well as the long-range electrostatic forces regulating protein–drug binding, and that the degree of this modulation is closely related to the electrostatic surface potential distribution on HSA as well as the charge properties of each TKI molecule. TKIs carrying higher net charges or localized charged moieties may experience stronger perturbations from the applied field due to enhanced electrostatic coupling with charged domains on HSA, whereas more neutral or hydrophobic molecules are comparatively less sensitive. This is similar to previous studies, which have shown that electric fields can induce dipole reorientation and charge redistribution within proteins, thereby altering their interaction patterns [55]. Friction force measurements further supported these findings: both loop friction and constant-load friction forces decreased substantially with field strength, indicating reduced energy dissipation and enhanced interfacial lubrication. The extent of friction reduction was drug-dependent, with highly hydrophobic TKI drugs such as radotinib and nilotinib showing a marked decrease. This highlights the important role of drug-specific binding patterns in regulating electric field sensitivity. Morphologically, AFM imaging revealed significant protein compaction and surface smoothing under electric field exposure. HSA average surface height decreased by 41–50%, while surface roughness reduced by 31–48%, consistent with field-driven conformational flattening and hydration shell reorganization [56]. These structural changes likely contribute to the observed mechanical modulation, by altering the steric and hydration-mediated components of interfacial forces.

Overall, our research findings indicate that an external electric field can serve as an effective means to dynamically regulate the mechanical properties and morphology of protein–drug interfaces. The synergistic reduction in adhesion force, specific/non-specific force, friction force, and surface roughness and average surface height indicates that electric fields can weaken binding affinity and promote the formation of smoother, more uniform interfaces. These effects are particularly pronounced in hydrophobic drug systems, as electric field-induced disruption of hydrophobic clusters triggers a stronger response. It should be noted that while our experimental design minimizes potential field-induced effects on the gold-coated cantilever and substrate, the absence of dedicated control measurements with unmodified cantilevers represents a limitation of this study. Nevertheless, previous AFM studies under similar conditions have shown negligible field effects on bare cantilever–substrate systems [57,58], supporting the interpretation that the observed variations primarily reflect changes in protein–protein interactions. From a broader perspective, these findings have important implications for the design of electrically responsive biomaterials, controlled drug delivery systems, and bioelectronic interfaces. By tuning interfacial interactions via external electric fields, it may be possible to regulate drug binding and release kinetics, enhance the biocompatibility of therapeutic interfaces, or create dynamic surfaces that respond to physiological cues. In the context of tyrosine kinase inhibitors (TKIs), the ability to modulate their interaction strength with plasma proteins such as human serum albumin (HSA) under electric fields may offer a novel strategy to optimize their pharmacokinetic behavior, improve systemic bioavailability, and enhance therapeutic efficacy, particularly in targeted or site-specific delivery scenarios. Moreover, our study highlights the utility of AFM-based nanomechanical techniques for elucidating fundamental interfacial phenomena in complex biological environments, offering a quantitative platform for the rational design of next-generation electro-responsive drug delivery systems.

## Figures and Tables

**Table 1:** Comparison of specific forces (*F_i_*) and non-specific forces (*F*_0_) between HSA molecules under different electric field strengths in the presence of different tyrosine kinase inhibitors (TKIs), based on >300 independent force-distance curves under each condition, calculated using the Poisson method from adhesion forces fitted by Gaussian fitting, reported as mean ± standard deviation (SD).

Sample	*F_i_*/*F*_0_ (pN)	0 (mV/mm)	20 (mV/mm)	40 (mV/mm)	60 (mV/mm)	80 (mV/mm)	100 (mV/mm)
Imatinib	*F_i_*	35.1 ± 0.5	33.4 ± 0.4	31.6 ± 0.2	28.9 ± 0.3	27.2 ± 0.5	25.2 ± 0.3
	*F*_0_	101 ± 2	90 ± 2	80 ± 1	71 ± 2	62 ± 2	52 ± 1
Bosutinib	*F_i_*	29.1 ± 0.4	27.0 ± 0.4	25.8 ± 0.6	24.7 ± 0.2	23.2 ± 0.3	21.3 ± 0.7
	*F*_0_	114 ± 1	103.7 ± 0.9	93 ± 2	81 ± 1	72 ± 2	64 ± 1
Dasatinib	*F_i_*	37.3 ± 0.8	35.6 ± 0.7	32.7 ± 0.7	30.3 ± 0.5	27.5 ± 0.4	26.0 ± 0.8
	*F*_0_	112 ± 1	103 ± 1	95 ± 2	85 ± 1	75 ± 2	67 ± 2
Nilotinib	*F_i_*	34.1 ± 0.6	32.0 ± 0.7	30.1 ± 0.5	28.2 ± 0.3	26.8 ± 0.4	24.5 ± 0.5
	*F*_0_	103 ± 1	91.4 ± 0.9	81 ± 2	70 ± 2	59 ± 1	49 ± 1
Ponatinib	*F_i_*	40.4 ± 0.7	36.9 ± 0.5	34.4 ± 0.6	31.5 ± 0.9	28.9 ± 0.5	25.4 ± 0.6
	*F*_0_	108 ± 1	100 ± 1	92.6 ± 0.9	84 ± 2	76 ± 3	67 ± 1
Radotinib	*F_i_*	42.1 ± 0.6	37.7 ± 0.5	34.6 ± 0.4	31.2 ± 0.7	27.4 ± 0.7	23.6 ± 0.4
	*F*_0_	116 ± 2	103 ± 1	93 ± 1	81 ± 2	68 ± 2	57 ± 2

## Data Availability

The data presented in this study are available on request from the corresponding author.

## Supplementary Materials

The following supporting information can be downloaded at: https://www.mdpi.com/article/10.3390/molecules30173558/s1, Figure S1: Schematic diagram of electrical stimulation device.
