# Supplementary figures and images for: Electric Field-Driven Modulation of Nanomechanical Interactions Between Tyrosine Kinase Inhibitors and Human Serum Albumin: Insights from AFM-Based Force Spectroscopy
Source: Molecules. 2025 Aug 30;30(17):3558. doi: 10.3390/molecules30173558 (PMC12430330; doi:10.3390/molecules30173558)

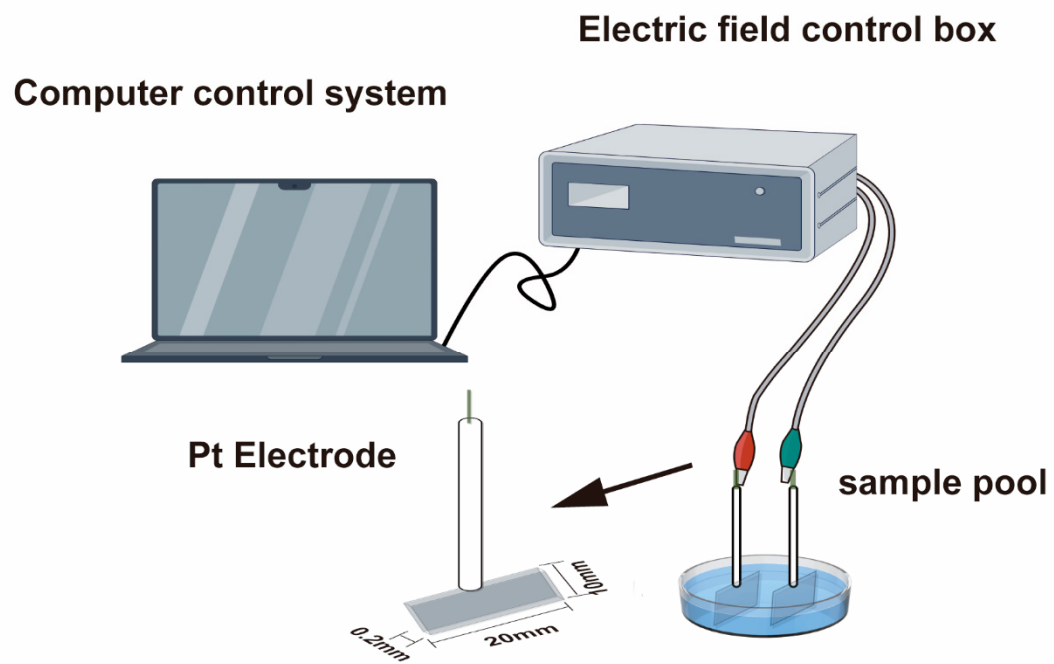

Figure S1: Schematic diagram of electrical stimulation device

Supplement: Supplementary file 1 [file molecules-30-03558-s001.zip › Supplementary Files.pdf]
